# Multiple Substance Use Patterns and Its Relationship with Imprisonment in a High-Risk Group of Iranian Adults

**DOI:** 10.34172/jrhs.2023.125

**Published:** 2023-09-29

**Authors:** Jalal Ale-Ebrahim, Sima Afrashteh, Leila Janani, Seyed Ahmad Seyed Alinaghi, Seyed Abbas Motevalian, Arezoo Kasavandi, Mahdi Sedgh Azar, Mansour Sajadipour, Behnam Farhoudi, Abbas Abbasi-Ghahramanloo

**Affiliations:** ^1^Department of Epidemiology, Iran University of Medical Sciences Tehran, Iran; ^2^Department of Biostatistics and Epidemiology, Faculty of Health and Nutrition, Bushehr University of Medical Sciences, Bushehr, Iran; ^3^Department of Biostatistics, School of Public Health, Iran University of Medical Sciences, Tehran, Iran; ^4^Iranian Research Center for HIV/AIDS, Iranian Institute for Reduction of High-Risk Behaviors, Tehran University of Medical Sciences, Tehran, Iran; ^5^Research Center for Addiction and High-Risk Behaviors (ReCARB), Psychosocial Health Research Institute, Iran University of Medical Sciences, Tehran, Iran; ^6^Department of Microbiology, Shahr-e-Qods Branch, Islamic Azad University, Tehran, Iran; ^7^Head of Behavioral Disease Counselling Center Boali, Vice Chancellor of Health, Shahid Beheshti University of Medical Sciences, Tehran, Iran; ^8^Department of Epidemiology, Tehran University of Medical Sciences, Tehran, Iran; ^9^Social Determinants of Health Research Center, Amiralmomenin Hospital, Tehran Medical Sciences Branch, Islamic Azad University, Tehran, Iran; ^10^Department of Public Health, School of Health, Ardabil University of Medical Sciences, Ardabil, Iran

**Keywords:** Substance use, Latent class analysis, Prisoners, Illicit drugs

## Abstract

**Background:** Substance use is recognized as an important factor associated with many diseases and premature deaths and the main risk factor for disability worldwide. This study aims to identify subgroups of substance use in adults and detect the effect of imprisonment on the membership of participants in latent classes of substance use.

**Study Design:** A cross-sectional study.

**Methods:** This study was performed on 930 adult people who were referred to behavioral health counseling centers in Tehran province. All participants completed some checklists and questionnaires. Data analysis was performed using chi-square, Fisher’s exact test, and latent class analysis (LCA).

**Results:** Four latent classes were identified, including non-users (58%), cigarette smokers (11.6%), users of low stigma substances (27.4%), and drug users (3.1%). After adjusting for other studied variables, having a history of imprisonment increased the odds of membership in the cigarette smoker class (Odds ratio [OR]=5.82, 95%, confidence interval [CI]: 3.19-10.63) and drug user class (OR=53.59, 95% CI: 18.00- 159.52) compared to non-user class. Among all participants, 84 (9.0%) had a history of imprisonment.

**Conclusion:** Results from the present study indicate that 30.5% of the participants fell under the user of the low-stigma substance or drug user group. Focusing on increasing prisoner’s knowledge of the dangers of using different substances and considering various programs for prisoners’ leisure time may help reduce substance use prevalence.

## Background

 Substance use refers to the illicit use of psychoactive substances or narcoticdrugs and is recognized as an important factor associated with many diseases and premature deaths and is the main risk factor for disability worldwide.^1,2^

 Substance use can affect a large number of organs and systems in the human body, leading to numerous medical problems and an increased risk of multiple diseases. For example, alcohol or drug use can impair a person’s self-care abilities, which may subsequently increase the risk of cardiovascular disease, cancers, road traffic accidents, liver disease, and disease complications.^3,4^ Decreased social welfare, health problems, mental disorders, and premature mortality are also some of the most important consequences of substance misuse.^3,5^

 It is estimated that the prevalence of substance use among adults shows an increasing trend. As such, the results of the Monitoring the Future study have indicated that the prevalence of substance use in this age group increased from 38.2% in 1999 to 49% in 2017.^6^

 The death epidemic from opioid use in the United States has turned into a serious health concern as the use of illicit drugs, psychoactive substances, and cocaine is particularly increasing in this country.^7^ In Europe, 11% of disability-adjusted life years are due to alcohol and illicit drugs.^8^ Moreover, the misuse of substances such as cannabis, amphetamines, opioids, and tobacco is increasing and is a major threat to public health in Asian countries.^9,10^ Opium has also been widely used for a long time in Iran, so opium use in this country is three times higher than the global average.^11,12^ It should be noted that the use of heroin and stimulants has also shown an increasing trend in Iran.^13^ Furthermore, the substance abuse mortality rate in Iran increased between 2014 and 2018.^2^

 Voluntary Counseling and Testing (VCT) was first developed in 1995 as a major tool for HIV/AIDS control and a viable means of preventing the disease spread.^13^ In Iran, behavioral health counseling centers provide services for patients with AIDS and other diseases, HIV counseling, diagnostic tests, and services aimed at harm reduction in substance use (e.g., syringe and needle exchange) and a condom to ensure protected intercourse.^14^

 The latent class analysis (LCA) provides a framework to identify heterogeneity within the population by analyzing the behavioral patterns of individuals so that the latent groups with similar patterns are identified in a set of observations.^15^

 This study aimed to identify subgroups of substance use in adults and detect the effect of imprisonment on the membership of participants in latent classes of substance use in people who were referred to behavioral health counseling centers using LCA.

## Methods

 This cross-sectional study was conducted on 930 adult people who were referred to behavioral health counseling centers in Tehran province from March 2018 to March 2020. Participants were selected by convenience sampling from three behavioral health counseling centers, including Bouali Counseling Center in Eastern Tehran, Darband Counseling Center in Northern Tehran, and Imam Khomeini hospital Counseling Center in the center of Tehran. All clients of these three counseling centers who were detected as having high-risk behaviors for HIV/AIDS were recruited into this study after being counseled by counselors and being referred to a laboratory for HIV testing. The interview was conducted after the test and before the announcement of the test results. First, the trained interviewers explained the purpose of the study to the participants, and if they were willing to participate in the study, they would start to be interviewed, after obtaining the informed consent. The format of the checklist used in this study was similar to the individual HIV test form. If the person had an acceptable literacy the checklist was given to the participant to answer the questions himself. Finally, the answers were checked by the interviewer, and unanswered questions were asked by the interviewer.

 The study was conducted using a checklist designed based on a form commonly used in VCT centers in Iran. The necessary data were collected by a questionnaire containing information on high-risk behaviors, including heterosexual and homosexual intercourse and the history of injecting drug users.

 To report some characteristics of participants and levels of engagement in each substance, we used descriptive statistics. In the next step, we performed LCA seven times using one to seven classes to find the best model. For model identification, each model has been fitted 20 times with different starting values. For the selection of the best model, some statistics were calculated and then compared across all models (seven models). These indices were likelihood-ratio statistic G2, the Akaike information criterion (AIC), the Bayesian information criterion (BIC), entropy, and the log-likelihood value. The lower value of G2, AIC, BIC, and the log-likelihood show a more optimal model fit; however, the higher value of entropy indicates more fitness of a model. For better identification, the interpretability of the results and parsimony of a model should be considered.

 Eight dichotomous observable variables (i.e., indicators) were used to detect latent subgroups of participants based on substance use. These variables were cigarette smoking, hookah smoking, alcohol, cannabis, ecstasy, amphetamine-type stimulants (ATS), opium, and heroin use. After identifying the optimal model, we conducted an LCA by considering covariates to detect the effect predictors of latent class membership. These covariates were age, gender, education, job, and imprisonment history. To investigate the effect of predictors, the non-user class was considered the reference class.

 To perform a simple statistical analysis, the chi-square and Fisher’s exact test were used by SPSS 16, and LCA was performed by using PROC LCA in SAS 9.2 software. In all analyses, *P *value < 0.05 was considered statistically significant.

## Results

 From 1090 distributed questionnaires, a total of 930 (85.3% response rate) were completed by the participants. In this study, the mean age of the participants was 32.09 ± 9.17 (range: 17-66) years old. Most of the participants were male (73.2%), and 9.0% of them had a history of imprisonment. Furthermore, 0.9% of the sample was illiterate, and only 9.8% were unemployed.

Table 1 presents the prevalence of different substance use based on the history of imprisonment. This table shows that some substances are more common than others. For example, the prevalence of cigarette smoking, hookah smoking, and alcohol use was obtained to be 42.5%, 30.9%, and 39.1%, respectively. On the other hand, ecstasy use was an uncommon substance among participants with a prevalence of 0.8%. Table 1 indicates a significant relationship between cigarette smoking, cannabis, ATS, opium, and heroin use with a history of imprisonment.

**Table 1:** Substance use by imprisonment in a sample of high-risk group of Iranian adults

**Variables**	**Total**		**Not imprisonment**		**Imprisonment**		***P***** value**
	**Number**	**Percent**	**Number**	**Percent**	**Number**	**Percent**	
Cigarette smoking (last month)							
No	535	57.5	519	61.3	16	19.0	0.001
Yes	395	42.5	327	38.7	68	81.0	
Hookah smoking (last month)							
No	643	69.1	584	69.0	59	70.2	0.819
Yes	287	30.9	262	31.0	25	29.8	
Alcohol use (last year)							
No	566	60.9	510	60.3	56	66.7	0.253
Yes	364	39.1	336	39.7	28	33.3	
Cannabis use (last year)							
No	856	92.0	786	92.9	70	83.3	0.002
Yes	74	8.0	60	7.1	14	16.7	
Ecstasy use (last year)							
No	923	99.2	840	99.3	83	98.8	0.486
Yes	7	0.8	6	0.7	1	1.2	
Amphetamine-type stimulants (last year)							
No	899	96.7	831	98.2	68	81.0	0.001
Yes	31	3.3	15	1.8	16	19.0	
Opium (last year)							
No	873	93.9	811	95.9	62	73.8	0.001
Yes	57	6.1	35	4.1	22	26.2	
Heroin (last year)							
No	896	96.3	838	99.1	58	69.0	0.001
Yes	34	3.7	8	0.9	26	31.0	

 Eight dichotomous variables were used to conduct LCA. The different measures of model selection are shown in Table 2. For the selection of the final model, we compared the values of G^2^, AIC, BIC, entropy, and the log-likelihood across seven models. According to model selection criteria and interpretability of the results, the four-class model was identified as the best model. Table 3 present the result of the LCA model for substance use pattern. This table includes the latent class prevalence and item response probabilities. The participants of this study were grouped into the non-user class (58%), cigarette smokers (11.6%), users of low-stigma substances (27.4%), and drug users (3.1%). Specifically, people in the non-user class had low probabilities of using all substances. Participants in the cigarette smoker class had a high probability of cigarette smoking (98.8%). It should be noted that the probability of hookah smoking was 34.8% among participants of this class. Moreover, individuals in the group of low-stigma substance users had a high probability of cigarette smoking (77.3%), hookah smoking (59.6%), and alcohol use (98.3%). Finally, among individuals in the drug user class, cigarette smoking (89.3%), alcohol (87.5%), cannabis (74.9%), ATS (64.8%), opium (70.5%), and heroin use (64.7%) had high probabilities. Table 3 indicates that among all substances, only ecstasy use had no important role in classifying the participants. In other words, the probability of using ecstasy was obtained to be under 50% in all latent classes.

**Table 2:** Comparison of LCA models with different latent classes based on model selection statistics

**Number of Latent Class**	**No. of estimated parameters**	**G**^2^	**df**	**AIC**	**BIC**	**Maximum** **log-likelihood**
1	8	257.34	247	773.34	812.02	-2626.91
2	17	294.46	238	328.46	410.66	-2395.47
3	26	175.10	229	227.10	352.82	-2335.79
4	35	129.64	220	199.64	368.88	-2313.06
5	44	92.75	211	180.75	393.50	-2294.62
6	53	77.25	202	183.25	439.51	-2286.87
7	62	69.18	193	193.18	492.97	-2282.84

*Note.* LCA: Latent class analysis; df: Degrees of freedom; AIC: Akaike information criterion; BIC: Bayesian information criterion.

**Table 3:** The four latent classes’ model of pattern of substance use among a sample of high-risk groups of Iranian adults

	**Non** **User**	**Cigarette** **smoker**	**User of low** **stigma substances**	**Drug** **user**
Latent class prevalence	0.580	0.116	0.274	0.031
Item-response probabilities				
Cigarette smoking (last month)	0.122	0.988	0.773	0.893
Hookah smoking (last month)	0.157	0.348	0.596	0.455
Alcohol use (last year)	0.159	0.028	0.983	0.875
Cannabis use (last year)	0.004	0.008	0.193	0.749
Ecstasy use (last year)	0.000	0.009	0.010	0.115
Amphetamine-type stimulants (last year)	0.002	0.082	0.010	0.648
Opium (last year)	0.010	0.137	0.064	0.705
Heroin (last year)	0.000	0.118	0.010	0.647

*Note*. The probability of a “No” response can be calculated by subtracting the item-response probabilities shown above from 1. Item-response probabilities > 0.5 in bold to facilitate interpretation.

 We found four significant predictors of latent class membership (Table 4), implying different distributions of latent class membership across these variables. With increasing age, the odds of being a user of low-stigma substance class decreased to 0.96. Being male increased the odds of membership in the cigarette smoker (OR = 4.24) and user of low-stigma substance (OR = 2.10) classes compared to the non-user class. Furthermore, being unemployed increased the odds of being in cigarette smoker (OR = 2.80), user of low-stigma substances (OR = 2.33), and drug use classes (OR = 9.39) relative to the “non-user” class. Moreover, having a history of imprisonment had the strongest effect on the membership of participants in different latent classes in this study and increased the odds of membership in the cigarette smoker (OR = 5.82) and drug use (OR = 53.59) classes compared to the non-user class.

**Table 4:** Predictors of membership in latent classes of substance use among Iranian adults

**Predictors**	**Cigarette smoker**	**User of low stigma substances**	**Drug user**	***P***** value**
	**OR (95% CI)**	**OR (95% CI)**	**OR (95% CI)**	
Age	1.02 (1.00, 1.04)	0.96 (0.94, 0.98)	0.99 (0.95, 1.04)	0.001
Gender (being male)	4.24 (2.13, 8.41)	2.10 (1.50, 2.96)	7.37 (0.77, 70.69)	0.001
Education (illiterate)	0.32 (0.06, 1.57)	0.84 (0.23, 3.01)	0.01 (0.00, 7.97)	0.272
Job (unemployment)	2.80 (1.56, 5.00)	2.33 (1.46, 3.73)	9.39 (4.02, 21.90)	0.001
Imprisonment history	5.82 (3.19, 10.63)	1.64 (0.84, 3.20)	53.59 (18.00, 159.52)	0.001

*Note.* Odds ratio; CI: Confidence interval; The reference group: Non user.

## Discussion

 The results of the present study showed four latent classes for patterns of substance use among Iranian adults who were referred to health counseling centers. The prevalence of non-users, cigarette smokers, users of low-stigma substances, and drug user classes were 58%, 11.6%, 27.4%, and 3.1%, respectively. Members of a non-user class displayed a lower probability of using all types of substances. The results of the present study suggest a significant co-occurrence of substance use among Iranian adults. As such, a high probability of using cigarettes, alcohol, cannabis, ATS, opium, and heroin was observed in the drug user class. Tzilos et al showed that daily marijuana use is associated with a significantly increased likelihood of opiate, cocaine, hallucinogen, inhalant, and tobacco use in the United States.^16^ The results of a review study also showed that more than 90% of cocaine and methamphetamine users report smoking.^17^ These findings demonstrated the considerable importance of noticing the co-occurrence nature of substance use in developing preventive interventions. Therefore, focusing on the co-incidence and co-using of different substances may effectively contribute to the reduction of substance use.

 Different person-centered studies have used different variables to identify subgroups of substance use among adults, and the results of some relatively similar studies can be discussed here. Liu et al identified five latent classes of substance use among lifetime cocaine users: Past 30-day tobacco use only (45%), past 30-day alcohol, marijuana, and tobacco use (31%), past 30-day tobacco, prescription of opioid and sedative use (13%), past 30-day cocaine, alcohol, marijuana, and tobacco use (9%), and past 30-day cocaine and multiple polysubstance use (2%).^18^ Another study in Australia revealed five latent classes for the pattern of polysubstance use: Alcohol only, alcohol and tobacco, cannabis, ecstasy, and licit drug use, cannabis, amphetamine derivatives, and illicit drug use, and sedative and alcohol use.^19^ Due to demographic differences and variations in substance use patterns, the results of substance use classification can be different in various countries. However, there are some levels of co-occurrence in substance use patterns in person-centered studies.

 The findings of the present study showed that the male gender significantly increased the odds of membership in cigarette smokers and users of low-stigma substances classes compared to non-user classes. Merlin et al. found that the female gender was associated with lower substance use.^20^ In another study, men were at higher risk of polysubstance use.^19^ Compared to men, Iranian women have less tendency toward substance use due to their socio-cultural background.^21^ In addition, men are more likely to use substances due to social norms and gender roles.^22^

 We found that unemployment increased the odds of a cigarette smoker, a user of low-stigma substances, and drug user membership compared to the non-user class. Generally, unemployment is associated with substance abuse in both men and women.^23^ Studies also have shown that recession and unemployment increase psychological stress, which can lead to illicit drug use.^24,25^ Melchior et al also indicated that an increased unemployment rate is associated with an increased rate of illicit drug use.^26^ Therefore, basic long-term control measures aiming at reducing unemployment should be established to subsequently reduce the prevalence of substance use.

 The present study showed that a history of imprisonment significantly increases the odds of being in the cigarette smoker and drug user classes compared to the non-user class. Studies in different countries showed that substance use is higher among prisoners than in the general population.^27,28^ The results of a study conducted in Iran showed that the prevalence of substance use in Iran’s prisons is magnificently high, and the history of imprisonment is an important risk factor for substance use.^29^ Yitayih et al also reported that incarcerated people have poor social support, making them more exposed to illicit drugs.^30^ Being in a weak social context with insufficient economic resources, being exposed to drugs in their place of residence, and stressful situations are important factors associated with substance use in people with a history of imprisonment.^31^ Therefore, harm reduction programs and interventions aimed at reducing the prevalence and complications of drug use in prisoners are of great importance.

 One of the study limitations was using a self-administered questionnaire, which could lead to the underestimation of the results. Additionally, this cross-sectional study was unable to explain the causal relationship between independent variables and substance use.

**Highlights**

- Four latent classes were identified, including non-users (58%), cigarette smoker (11.6%), users of low-stigma substances (27.4%), and drug user (3.1%).
- Alcohol use was more common among participants.
- Having a history of imprisonment increased the odds of membership in the cigarette smoker and drug user classes.
- Among all participants, 84 (9.0%) had a history of imprisonment.

## Conclusion

 In the present study, we evaluated the prevalence and pattern of substance use among a sample of Iranian adults who were referred to behavioral health counseling centers. We also evaluated the impact of imprisonment as well as age, gender, education, and job status on membership in different latent classes identified by the LCA. It was found that although a small number of participants are in the drug user class, the probability of using most substances is quite high in this class. We found that a history of imprisonment increases the odds of being in cigarette smoker and drug user classes compared to non-user classes. Consequently, focusing on educational interventions in prisons could help prisoners to increase their knowledge of the dangers of using different substances. In addition, considering various programs for prisoners’ leisure time may help reduce substance use among these adults.

## Acknowledgments

 We would like to thank the Iranian Research Center for HIV/AIDS (IRCHA) affiliated with the Tehran University of Medical Sciences (TUMS) and the prison staff who assisted with data collection.

## Authors’ Contribution

**Conceptualization:** Jalal Ale-Ebrahim, Leila Janani, Seyed Ahmad Seyed Alinaghi, Seyed Abbas Motevalian, Abbas Abbasi-Ghahramanloo.

**Data curation:** Jalal Ale-Ebrahim, Leila Janani, Seyed Ahmad Seyed Alinaghi, Seyed Abbas Motevalian, Arezoo Kasavandi, Mahdi Sedgh Azar, Mansour Sajadipour, Behnam Farhoudi, Abbas Abbasi-Ghahramanloo.

**Formal analysis:** Jalal Ale-Ebrahim, Sima Afrashteh, Abbas Abbasi-Ghahramanloo.

**Funding acquisition:** Jalal Ale-Ebrahim and Seyed Abbas Motevalian

**Investigation:** Jalal Ale-Ebrahim, Leila Janani, Seyed Ahmad Seyed Alinaghi, Seyed Abbas Motevalian, Arezoo Kasavandi, Mahdi Sedgh Azar, Mansour Sajadipour, Behnam Farhoudi, Abbas Abbasi-Ghahramanloo.

**Methodology:** Jalal Ale-Ebrahim, Sima Afrashteh, Leila Janani, Seyed Abbas Motevalian, Abbas Abbasi-Ghahramanloo.

**Project administration:** Jalal Ale-Ebrahim, Seyed Abbas Motevalian, Abbas Abbasi-Ghahramanloo.

**Resources:** Jalal Ale-Ebrahim, Seyed Abbas Motevalian, Arezoo Kasavandi, Mahdi Sedgh Azar, Mansour Sajadipour, Behnam Farhoudi.

**Software:** Sima Afrashteh and Abbas Abbasi-Ghahramanloo.

**Supervision:** Leila Janani, Seyed Ahmad Seyed Alinaghi, Seyed Abbas Motevalian, Abbas Abbasi-Ghahramanloo.

**Validation:** Jalal Ale-Ebrahim, Leila Janani, Seyed Ahmad Seyed Alinaghi, Seyed Abbas Motevalian, Abbas Abbasi-Ghahramanloo.

**Visualization:** Jalal Ale-Ebrahim, Leila Janani, Seyed Ahmad SeyedAlinaghi, Seyed Abbas Motevalian, Abbas Abbasi-Ghahramanloo.

**Writing–original draft:** Jalal Ale-Ebrahim, Sima Afrashteh, Abbas Abbasi-Ghahramanloo

**Writing–review & editing:** Jalal Ale-Ebrahim, Sima Afrashteh, Leila Janani, Seyed Ahmad Seyed Alinaghi, Seyed Abbas Motevalian, Arezoo Kasavandi, Mahdi Sedgh Azar, Mansour Sajadipour, Behnam Farhoudi, Abbas Abbasi-Ghahramanloo.

## Competing Interests

 The authors declare that they have no competing interests.

## Funding

 None.

## References

[1] Saikia N, Debbarma B (2020). The socioeconomic correlates of substance use among male adults in Northeast India. Clin Epidemiol Glob Health.

[2] Shahbazi F, Mirtorabi D, Ghadirzadeh MR, Hashemi-Nazari SS (2018). Analysis of mortality rate of illicit substance abuse and its trend in five years in Iran, 2014-2018. Addict Health.

[3] Wu LT, Zhu H, Ghitza UE (2018). Multicomorbidity of chronic diseases and substance use disorders and their association with hospitalization: results from electronic health records data. Drug Alcohol Depend.

[4] Sunil Kumar D, Thomas JJ, Mohandas A, Chandana H, George PS, Narayana Murthy MR (2020). Prevalence of substance use and awareness about its ill effects among people residing in a rural village in Chamarajanagara district, Karnataka. Clin Epidemiol Glob Health.

[5] Tsai AC, Alegría M, Strathdee SA (2019). Addressing the context and consequences of substance use, misuse, and dependence: a global imperative. PLoS Med.

[6] Schulenberg JE, Johnston LD, O’Malley PM, Bachman JG, Miech RA, Patrick ME. Monitoring the Future National Survey Results on Drug Use, 1975-2016: Volume II, College Students and Adults Ages 19-55. Institute for Social Research, The University of Michigan; 2017.

[7] Kariisa M, Scholl L, Wilson N, Seth P, Hoots B (2019). Drug overdose deaths involving cocaine and psychostimulants with abuse potential - United States, 2003-2017. MMWR Morb Mortal Wkly Rep.

[8] Lievens D, Vander Laenen F, Christiaens J (2014). Public spending for illegal drug and alcohol treatment in hospitals: an EU cross-country comparison. Subst Abuse Treat Prev Policy.

[9] Dargan PI, Wood DM (2012). Recreational drug use in the Asia Pacific region: improvement in our understanding of the problem through the UNODC programmes. J Med Toxicol.

[10] Chun J (2020). Public health threat of tobacco and substance use in Asia: an introduction to the theme issue. J Psychoactive Drugs.

[11] Moradinazar M, Najafi F, Jalilian F, Pasdar Y, Hamzeh B, Shakiba E (2020). Prevalence of drug use, alcohol consumption, cigarette smoking and measure of socioeconomic-related inequalities of drug use among Iranian people: findings from a national survey. Subst Abuse Treat Prev Policy.

[12] Shahbazi F, Mirtorabi D, Ghadirzadeh MR, Shojaei A, Hashemi Nazari SS (2020). Years of life lost (YLL) due to substance abuse in Iran, in 2014-2017: global burden of disease 2010 method. Iran J Public Health.

[13] Ebrahimi M, Jalilian F, Ashtarian H, Heidari Z, Rajati F (2019). The role of integrative model of behavioral prediction in voluntary counseling of individuals with sexual high-risk behavior. J Educ Health Promot.

[14] Hajabdolbaghi M, Ghafarzadeh M, Bayat Jozani Z, Mohraz M, Farrokhi M, Foroughi M (2018). Situational analysis on voluntary counseling and confidential testing in HIV/AIDS patients referred to behavioral center in lmam Khomeini hospital, Tehran, Iran. Int J Epidemiol Res.

[15] Petersen KJ, Qualter P, Humphrey N (2019). The application of latent class analysis for investigating population child mental health: a systematic review. Front Psychol.

[16] Tzilos GK, Reddy MK, Caviness CM, Anderson BJ, Stein MD (2014). Getting higher: co-occurring drug use among marijuana-using emerging adults. J Addict Dis.

[17] Agrawal A, Budney AJ, Lynskey MT (2012). The co-occurring use and misuse of cannabis and tobacco: a review. Addiction.

[18] Liu Y, Elliott AL, Serdarevic M, Leeman RF, Cottler LB (2019). A latent class analysis of the past-30-day substance use patterns among lifetime cocaine users: findings from a community sample in North Central Florida. Addict Behav Rep.

[19] Quek LH, Chan GC, White A, Connor JP, Baker PJ, Saunders JB (2013). Concurrent and simultaneous polydrug use: latent class analysis of an Australian nationally representative sample of young adults. Front Public Health.

[20] Merline AC, O’Malley PM, Schulenberg JE, Bachman JG, Johnston LD (2004). Substance use among adults 35 years of age: prevalence, adulthood predictors, and impact of adolescent substance use. Am J Public Health.

[21] Rahimian Boogar I, Tabatabaee SM, Tosi J (2014). Attitude to substance abuse: do personality and socio-demographic factors matter?. Int J High Risk Behav Addict.

[22] Birhanu AM, Bisetegn TA, Woldeyohannes SM (2014). High prevalence of substance use and associated factors among high school adolescents in Woreta town, Northwest Ethiopia: multi-domain factor analysis. BMC Public Health.

[23] Teixidó-Compañó E, Espelt A, Sordo L, Bravo MJ, Sarasa-Renedo A, Indave BI (2018). Differences between men and women in substance use: the role of educational level and employment status. Gac Sanit.

[24] Nagelhout GE, Hummel K, de Goeij MCM, de Vries H, Kaner E, Lemmens P (2017). How economic recessions and unemployment affect illegal drug use: A systematic realist literature review. Int J Drug Policy.

[25] Compton WM, Gfroerer J, Conway KP, Finger MS (2014). Unemployment and substance outcomes in the United States 2002-2010. Drug Alcohol Depend.

[26] Melchior M, Chollet A, Elidemir G, Galéra C, Younès N (2015). Unemployment and substance use in young adults: does educational attainment modify the association?. Eur Addict Res.

[27] Mundt AP, Baranyi G, Gabrysch C, Fazel S (2018). Substance use during imprisonment in low- and middle-income countries. Epidemiol Rev.

[28] Fazel S, Yoon IA, Hayes AJ (2017). Substance use disorders in prisoners: an updated systematic review and meta-regression analysis in recently incarcerated men and women. Addiction.

[29] Moradi G, Darvishi S, Asaadi L, Azimian Zavareh F, Gouya MM, Tashakorian M (2020). Patterns of drug use and related factors among prisoners in Iran: results from the national survey in 2015. J Prim Prev.

[30] Yitayih Y, Abera M, Tesfaye E, Mamaru A, Soboka M, Adorjan K (2018). Substance use disorder and associated factors among prisoners in a correctional institution in Jimma, Southwest Ethiopia: a cross-sectional study. BMC Psychiatry.

[31] Binswanger IA, Nowels C, Corsi KF, Glanz J, Long J, Booth RE (2012). Return to drug use and overdose after release from prison: a qualitative study of risk and protective factors. Addict Sci Clin Pract.

